# Cancer cell-derived von Willebrand factor enhanced metastasis of gastric adenocarcinoma

**DOI:** 10.1038/s41389-017-0023-5

**Published:** 2018-01-24

**Authors:** Ai-jun Yang, Min Wang, Yan Wang, Wei Cai, Qiang Li, Ting-ting Zhao, Li-han Zhang, Katie Houck, Xu Chen, Yan-ling Jin, Ji-ying Mu, Jing-fei Dong, Min Li

**Affiliations:** 1 Institute of Integrated Traditional Chinese and Western Medicine, School of Basic Medical Sciences, Lanzhou University, Lanzhou, China; 2 Institute of Pathology, School of Basic Medical Sciences, Lanzhou University, Lanzhou, China; 3 Gansu Provincial Hospital, Lanzhou, China; 4 The First Affiliated Hospital of Lanzhou University, Lanzhou, China; 5 Bloodworks Research Institute, Seattle, Washington, USA; 6 Division of Hematology, Department of Medicine, University of Washington School of Medicine, Seattle, Washington, USA; 7 Key Laboratory of Preclinical Study for New Drugs of Gansu Province, Lanzhou University, Lanzhou, China

## Abstract

Cancer prognosis is poor for patients with blood-borne metastasis. Platelets are known to assist cancer cells in transmigrating through the endothelium, but ligands for the platelet-mediated cancer metastasis remain poorly defined. von Willebrand factor (vWF) is a major platelet ligand that has been widely used as a biomarker in cancer and associated inflammation. However, its functional role in cancer growth and metastasis is largely unknown. Here we report that gastric cancer cells from patients and cells from two well-established gastric cancer lines express vWF and secrete it into the circulation, upon which it rapidly becomes cell-bound to mediate cancer-cell aggregation and interaction with platelets and endothelial cells. The vWF-mediated homotypic and heterotypic cell–cell interactions promote the pulmonary graft of vWF-overexpressing gastric cancer BGC823 cells in a mouse model. The metastasis-promoting activity of vWF was blocked by antibodies against vWF and its platelet receptor GP Ibα. It was also reduced by an inhibitory siRNA that suppresses vWF expression. These findings demonstrate a causal role of cancer-cell-derived vWF in mediating gastric cancer metastasis and identify vWF as a new therapeutic target.

## Introduction

Metastasis is a major cause of cancer-related death, and its prevention is a significant challenge for efficient cancer treatments^1^. Blood-borne cancer metastasis occurs frequently, but the processes of its initiation and progression remain poorly defined.

Platelets play a key role in cancer development and metastasis^2^ and are often regarded as a “death ally” of cancer^1^. Cancer cells from multiple origins stimulate platelets to produce platelet-derived growth factor and matrix metalloprotease 2 to propagate inflammation^3^. They have also been widely reported to secrete platelet agonists such as adenosine diphosphate^4^ and thromboxane A2^5^ to induce platelet aggregation, which is often considered an early event in blood-borne cancer metastasis^2,6^. Consistently with these observations, antagonists to the platelet receptors integrin αIIbβ_3_ and glycoprotein Ib-IX-V complex have been reported to reduce cancer growth and metastasis^7,8^. However, the molecules that mediate the platelet-cancer interaction remains a matter of speculation. von Willebrand factor (vWF) is one of the major platelet adhesion ligands that could potentially regulate cancer development and metastasis.

vWF is the largest multimeric glycoprotein in human blood. It is thought to be exclusively synthesized in endothelial cells and megakaryocytes/platelets^9–11^. It tethers circulating platelets to the subendothelial matrix exposed at the site of vascular injury, but it also promotes platelet adhesion to endothelial cells in disease states^12^. Upon synthesis, pro-vWF monomers dimerize through C-terminal disulfide bonds^13^. A variable number of dimers then multimerize through N-terminal disulfide bonds^2,4–6^ after the cleavage of a large propeptide^3,7,8,14^. Newly synthesized vWF multimers are either constitutively released or stored in the Weibel–Palade bodies of endothelial cells and in the α-granules of megakaryocytes and platelets^15,16^. The stored vWF is enriched in ultra-large multimers^17^ and is released in response to inflammatory and ischemic injuries^18,19^. The plasma level of vWF is therefore a widely used marker for endothelial perturbation and propensity for thrombosis and thromboembolism^20,21^. Plasma vWF is significantly elevated in patients with cancer^15,22–25^. A high level of plasma vWF is associated not only with the development of cancer-associated thrombosis^26^, but also with the degree of malignancy, the rate of metastasis^27^, and cancer prognosis^28,29^. However, how vWF regulates cancer development and metastasis remains unknown. Furthermore, elevated levels of plasma vWF found in a persistent inflammatory state associated with cancer are often considered to come from perturbed endothelial cells and activated platelets, but osteosarcoma cells have been found to also express vWF^28,30^. Here we report the results of a study designed to detect vWF expression in gastric cancer cells and to examine a role of cancer-cell-derived vWF in promoting gastric cancer development and metastasis.

## Results

### Plasma levels and tissue expression of vWF in patients

The clinical cohort included 110 patients recruited from the First Affiliated Hospital of Lanzhou University between 2011 and 2014. The patients were diagnosed with either intraepithelial neoplasia (*n* = 16, 14.5%) or gastric adenocarcinoma (*n* = 94, 85.5%) by histological examinations of cancer tissues from biopsy or surgery (Table 1).

**Table 1:** Clinical characteristic of patients included in the study

Characteristics	Number (%)
Median age in years	58.4 ± 11.1
Male sex (%)	69 (73.4)
*Cancer location*	
Low stomach	50 (53.2)
Middle stomach	14 (14.9)
Upper stomach	22 (23.4)
Whole stomach	8 (8.5)
*Level of differentiation by histology*	
Well differentiated	26 (27.7)
Moderately differentiated	46 (48.9)
Poorly differentiated	22 (23.4)

**Fig. 1:**
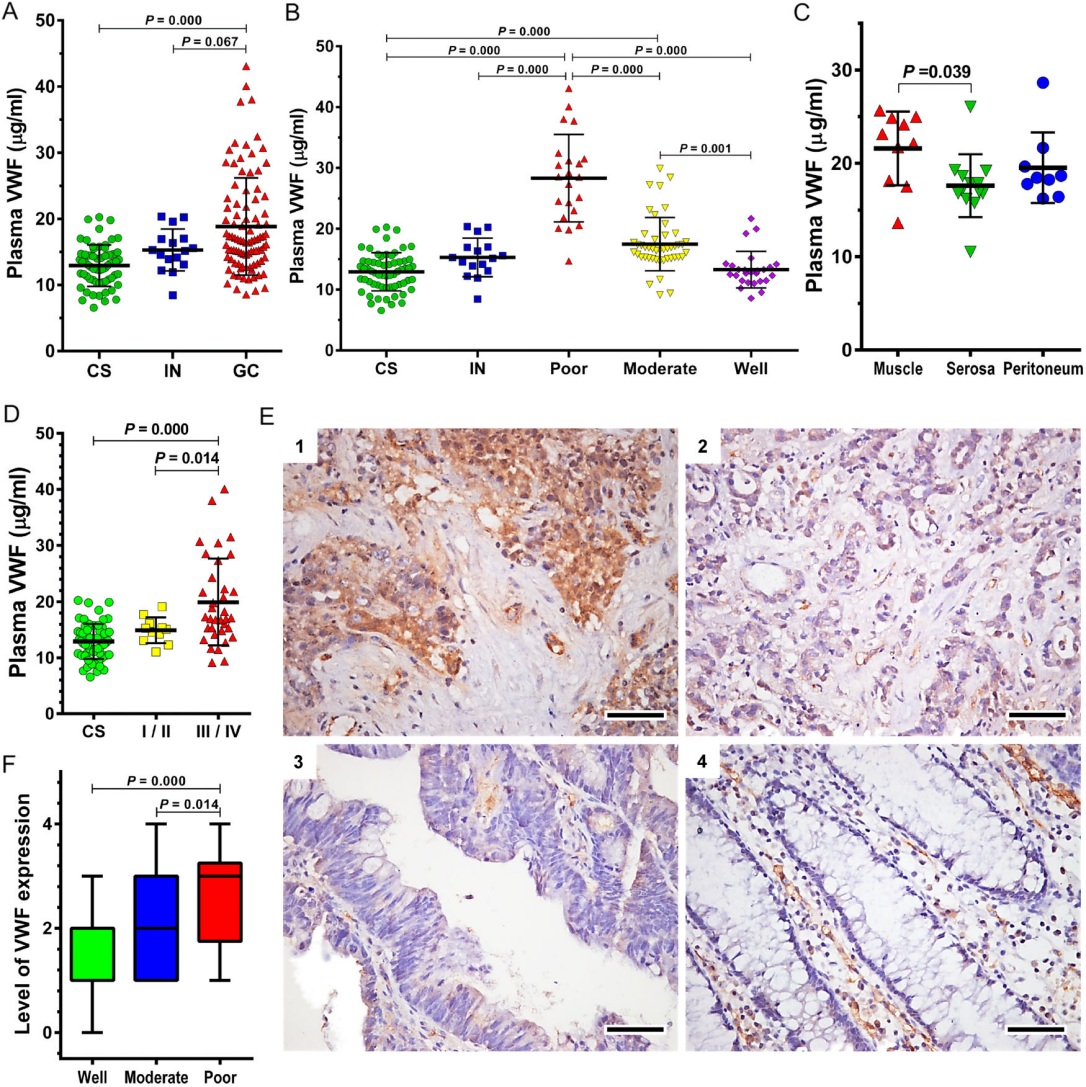
vWF in plasma and cancer cells of patients. **a** Plasma levels of vWF in heathy subjects (CS, *n* = 67) and in patients with intraepithelial neoplasia (IN, *n* = 16) or gastric cancer (GC, *n* = 94). Plasma vWF levels among patients grouped as having poorly, moderately, and well differentiated cancers (**b**), on the types of local invasion (**c**), and on international TNM developmental stages (**d**). Examples of vWF expression in gastric cancer tissues (**e**, dark brown stain, bar = 50 μm, (1): poorly differentiated GC, (2): moderately differentiated GC, (3): poorly differentiated GC, and (4): gastric mucosal tissue). **f** Quantitative comparisons of vWF expression in cancer cells among patients with well, moderately, and poorly differentiated gastric cancer (*n* = 94). Quantitative data present in **a**–**d**, **f** are analyzed by one-way ANOVA and Tukey HSD test

The plasma levels of vWF antigen were 12.92 μg/mL, 15.29 μg/mL, and 18.84 μg/mL for healthy controls, patients with intraepithelial neoplasia, and patients with gastric adenocarcinoma (Fig. 1a). Patients with poorly differentiated gastric adenocarcinoma had the highest levels of plasma vWF (Fig. 1b). A Pearson correlation analysis suggested an inverse relationship between the degree of cell differentiation and the plasma levels of vWF (*r* = −0.719, *p* < 0.01). While no difference was found between patients with lymph node metastasis and without (data not shown), the plasma levels of vWF were higher in patients with muscle-invasive cancer than in those with serosa-invasive cancer or peritoneal disseminated cancer (Fig. 1c). vWF was also higher in patients with late stages III or IV of cancer based on the TNM classification^29^, than in those with early stages I or II (Fig. 1d).

Immunocytochemistry detected vWF both in endothelial cells and in the gastric adenocarcinoma cells from all 94 patients with adenocarcinoma, as compared to exclusive vWF staining in endothelial cells of normal gland cells (Fig. 1e). The vWF expression quantified by antibody binding intensity was stronger in poorly differentiated cancerous tissues than in moderately differentiated and well-differentiated cancer cells (Fig. 1f). Regression analysis further suggested that vWF expression in cancer cells was associated with levels of cell differentiation (*r* = −0.4256, *p* < 0.05).

**Fig. 2:**
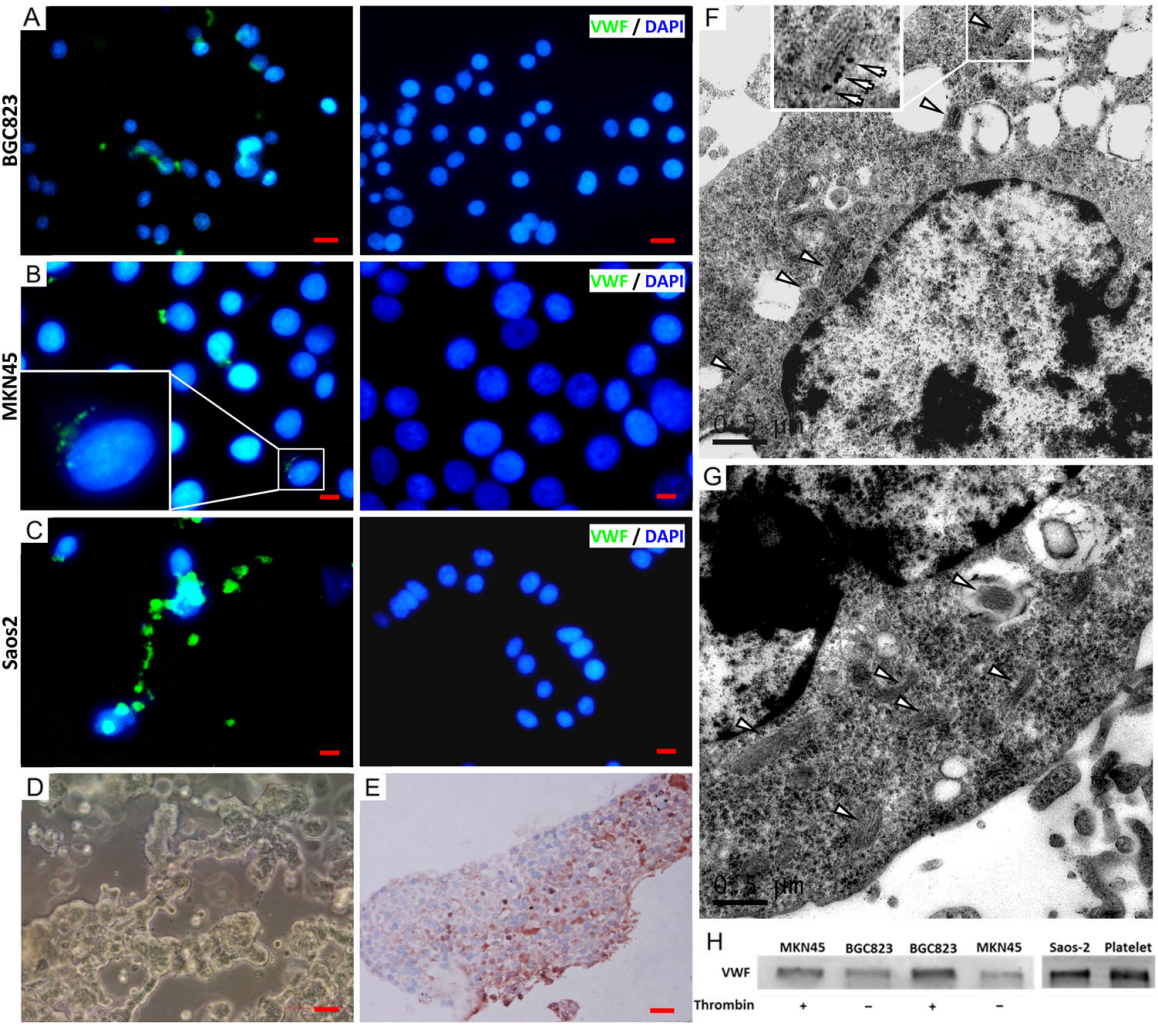
vWF expression in cancer cells. Clonal cells from the gastric cancer lines BGC823 (**a**), MKN45 (**b**), and the osteosarcoma line Saos2 (**c**) were stained with a vWF antibody (green, left panels) and counter-stained with DAPI (blue) or with non-immune IgG (right panels, **a**: bar = 50 μm, **b**, **c**: bar = 20 μm; each image is a representative of three independent experiments). BGC823 cells form cancerous masses after 20 days in a 3D-metrigel culture system (**d**, bar = 100 μm) and a BGC823 mass is positive for vWF (**e**, bar = 20 μm; images are representative of three independent experiments). Immunoelectron microscopic images of BGC823 cells (**f**) and HUVECs (**g**), showing Weibel–Palade body-like structures (arrow head) that are stained positive for vWF (bar = 0.5 μM). **h** Immunoblots of vWF in the culture supernatants from BGC823 and MKN45 cells before and after cells were stimulated with thrombin (0.5 U/mL). Lysates from the osteocarcinoma cell line Saos2 and human platelets were examined as controls (the blot is a representative of three separate cell preparations)

Consistently with findings in patients, vWF was detected in cells from the human gastric adenocarcinoma lines BGC823 (Fig. 2a) and MKN45 (Fig. 2b). However, the level of intracellular vWF was lower in these clonal cells than in primary cancer tissue from biopsy and surgery (data not shown). The reason for this phenotypic difference between the primary and clonal gastric cancer cells remains to be investigated, but its presence made the study of the biological activity of gastric cancer-derived vWF using the clonal cells difficult and non-representative. To address this concern, BGC823 cells used in vitro and in mouse experiments were transfected with human vWF cDNA to restore their vWF expression. These vWF-transfected cells were termed “vWF-overexpressing” to distinguish them from the parental BGC823 cells. As a control, vWF was also detected in human osteosarcoma Saos2 cells (Fig. 2c), consistent with a previous report^30^. BGC823 cells cultured in a 3D matrigel system formed cell masses (Fig. 2d) that were stained positive for vWF (Fig. 2e). Immunoelectron microscopy identified vWF primarily in Weibel–Palade body-like structures of BGC823 cells (Fig. 2f), similar to those found in HUVECs (Fig. 2g). Both MKN45 and BGC823 cells secreted vWF (Fig. 2h), but the vWF secretion was enhanced by the serine protease thrombin, which is known to induce endothelial cells to secret vWF^31^. We chose thrombin to induce vWF secretion from BGC823 and MKN45 cells for two reasons. First, vWF in these cells formed Weibel–Palade body-like structures (Fig. 2f) similar to those found in endothelial cells (Fig. 2g), suggesting that vWF is stored in granules of BGC823 cells. Second, the expression of the thrombin receptor PAR-1 has been reported in gastric and other cancers^32, 33^.

### Enhancement of cancer cell adhesion by vWF

We have shown that plasma vWF was significantly increased in patients with gastric cancer and that its levels were associated with the stage of cancer-cell differentiation and the rate of metastasis. We have also shown that gastric cancer cells synthesized and released vWF from unstimulated cells, but the secretion was enhanced by thrombin, suggesting that vWF was secreted through constitutive and induced pathways (the latter from the Weibel–Palade body-like granules), similar to those defined in endothelial cells^28, 30^. We next investigated whether vWF regulated the development and metastasis of gastric cancer using several complementary approaches.

**Fig. 3:**
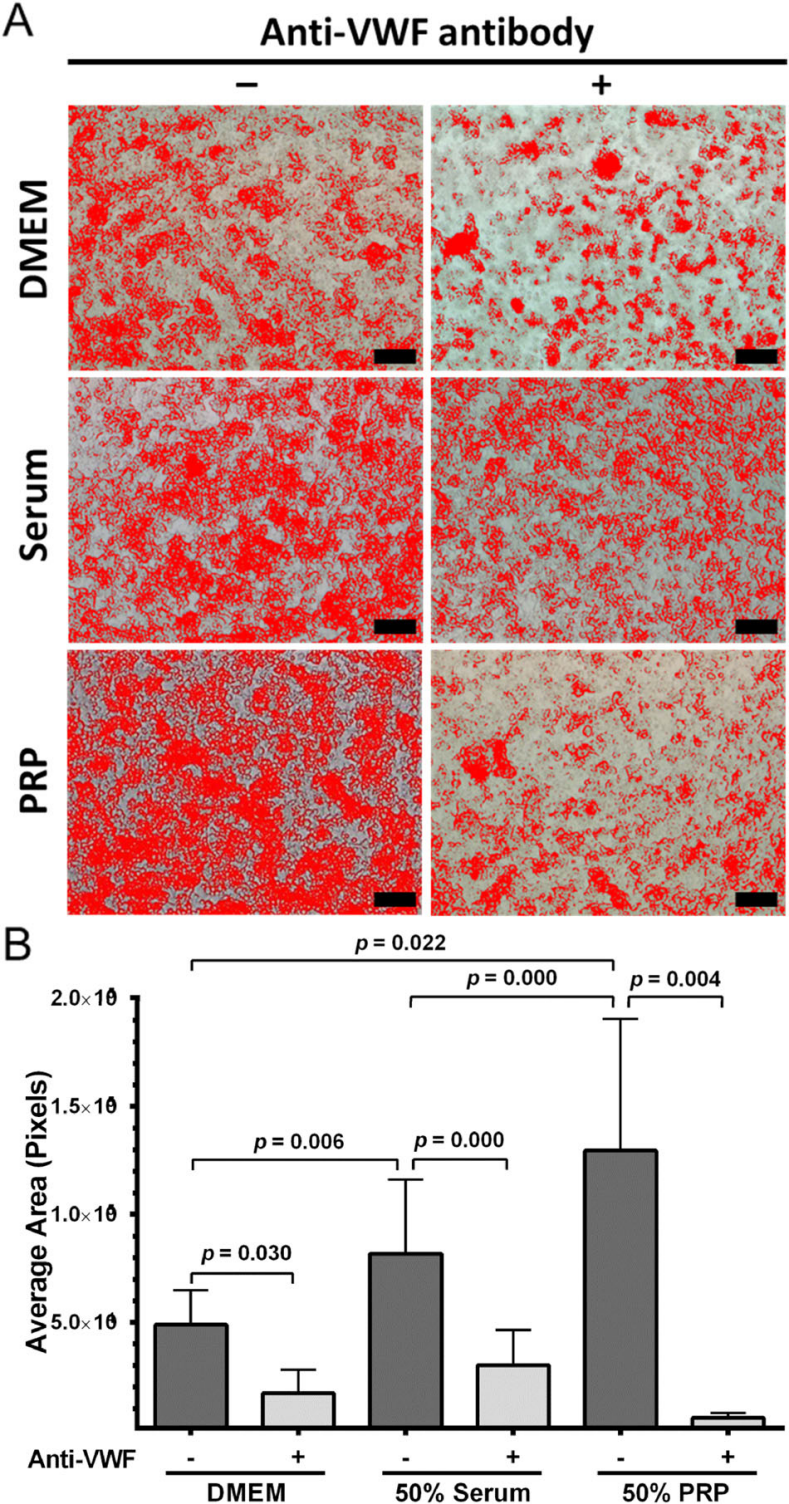
vWF mediates cancer cell aggregation. **a** Representative phase contrast images of homotypic aggregation of BGC823 cells in serum-free medium (top panel, bar = 200 μm) or in the medium containing 50% serum (middle panel, bar = 200 μm) or in medium containing 50% PRP (low panel, bar = 200 μm). The cells are marked with artificial color assigned by Image-Pro Plus 6.0 software. **b** A summary of three independent experiments (paired *t-*test)

**Fig. 4:**
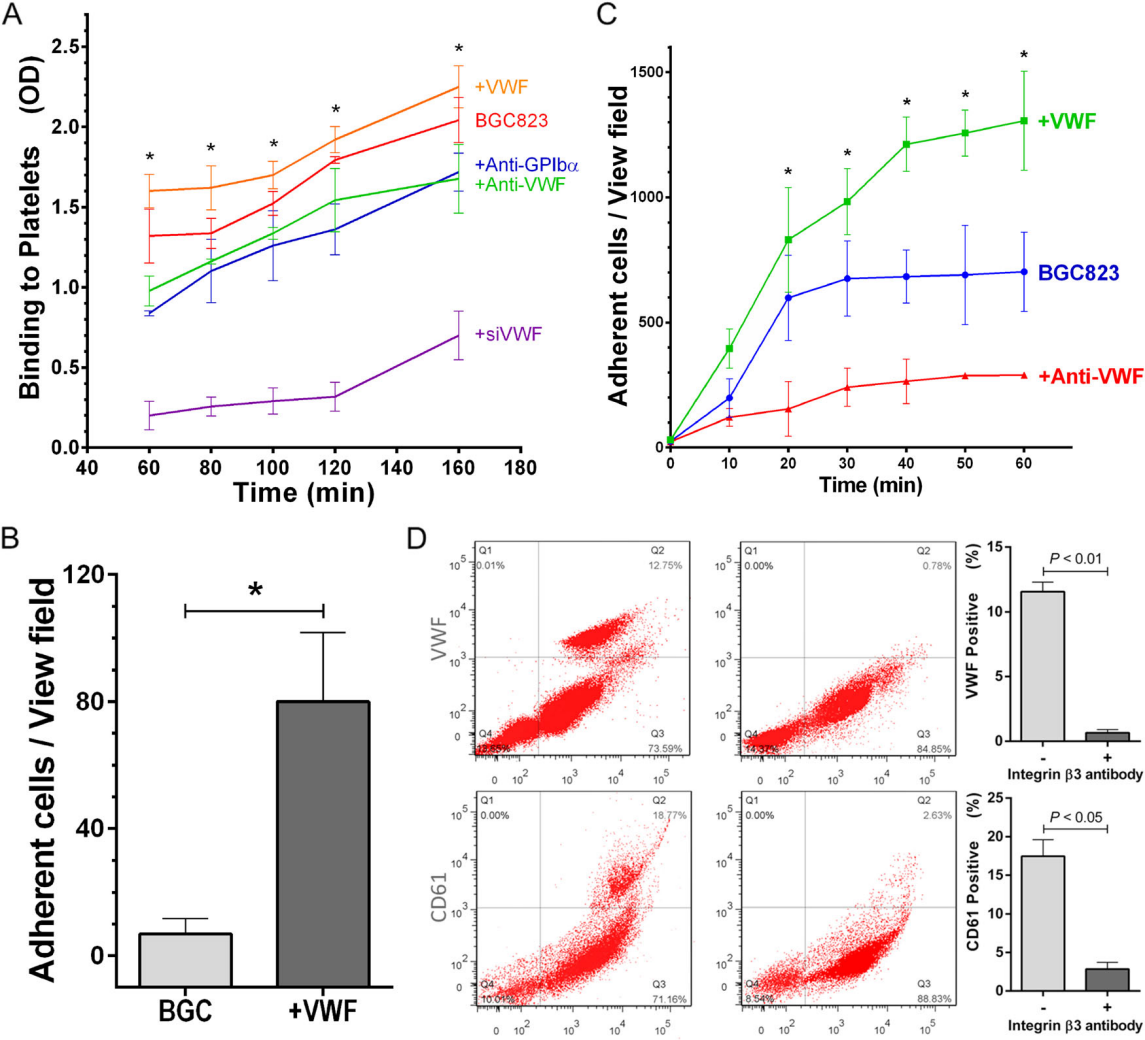
vWF enhances cancer cell adhesion to HUVECs and platelets. **a** Time course of BGC823 cells adherent to immobilized platelets as measured by MTT in the presence or absence of recombinant human vWF, an anti-human vWF antibody, or an anti-human GP Ibα antibody. BGC823 cells transiently transfected with an inhibitory vWF siRNA were identically tested (*n* = 3, one-way ANOVA, *p < 0.05 vs. untreated cells). **b** BGC823 cells were perfused over adherent platelets under a flowing condition in the presence or absence of recombinant human vWF (*n* = 3, Student’s *t-*test *p < 0.05). **c** The number of BGC823 cells adherent to cultured HUVECs in the presence or absence of either an anti-human vWF antibody or recombinant human vWF (*n* = 3, one-way ANOVA, *p < 0.05 vs. untreated cells). **d** The detection of surface-bound vWF on vWF-overexpressing BGC823 cells by flow cytometry. An anti-β3 integrin antibody reduces the surface-bound vWF (top panel, *n* = 3, Student’s *t*-test) and platelets (identified by an anti-CD61 antibody) bound to BGC823 cells (bottom panel, *n* = 3, Student's *t-*test). The values were presented after non-specific binding from an isotype control antibody was subtracted

First, BGC823 cells aggregated in a serum-free medium and the aggregation was blocked by a vWF antibody (Fig. 3a), suggesting that it depends on cell-derived vWF. However, the aggregation of BGC823 cells was enhanced by serum and further increased with platelet-rich plasma (PRP) in a vWF-dependent manner (Fig. 3), strongly indicating that plasma vWF and platelets enhanced the cancer cell aggregation. Second, BGC823 cells adhered to platelets and the adhesion was blocked by antibodies to vWF and its receptor GP Ib (Fig. 4a). Furthermore, the adhesion of BGC823 cells to platelets was reduced when they were transfected with a small interfering RNA (siRNA) that inhibited vWF synthesis, and the reduction was reversed by recombinant human vWF (Fig. 4a). Third, BGC823 cells adhered to human platelets under venous shear stress of 2 dynes/cm^2^, and the adhesion was increased by the addition of recombinant human vWF to the culture medium (Fig. 4b). Finally, BGC823 cells adhered to cultured endothelial cells in the absence of platelets (Fig. 4c). The adhesion was blocked by an anti-vWF antibody and was enhanced by recombinant human vWF. The finding that BGC823 cells interacted with endothelial cells in the absence of exogenous vWF suggests that the interaction was mediated by vWF secreted from BGC823 cells. It further indicates that, upon secretion, vWF becomes surface-bound to mediate the cell–cell interaction. This notion is supported by the finding that an anti-integrin β3 antibody eliminated surface-bound vWF on BGC823 cells (Fig. 4d, top panel) and blocked platelet binding to BGC823 cells (Fig. 4d, bottom panel).

### Increased adhesiveness of cancer cells by overexpressing vWF

**Fig. 5:**
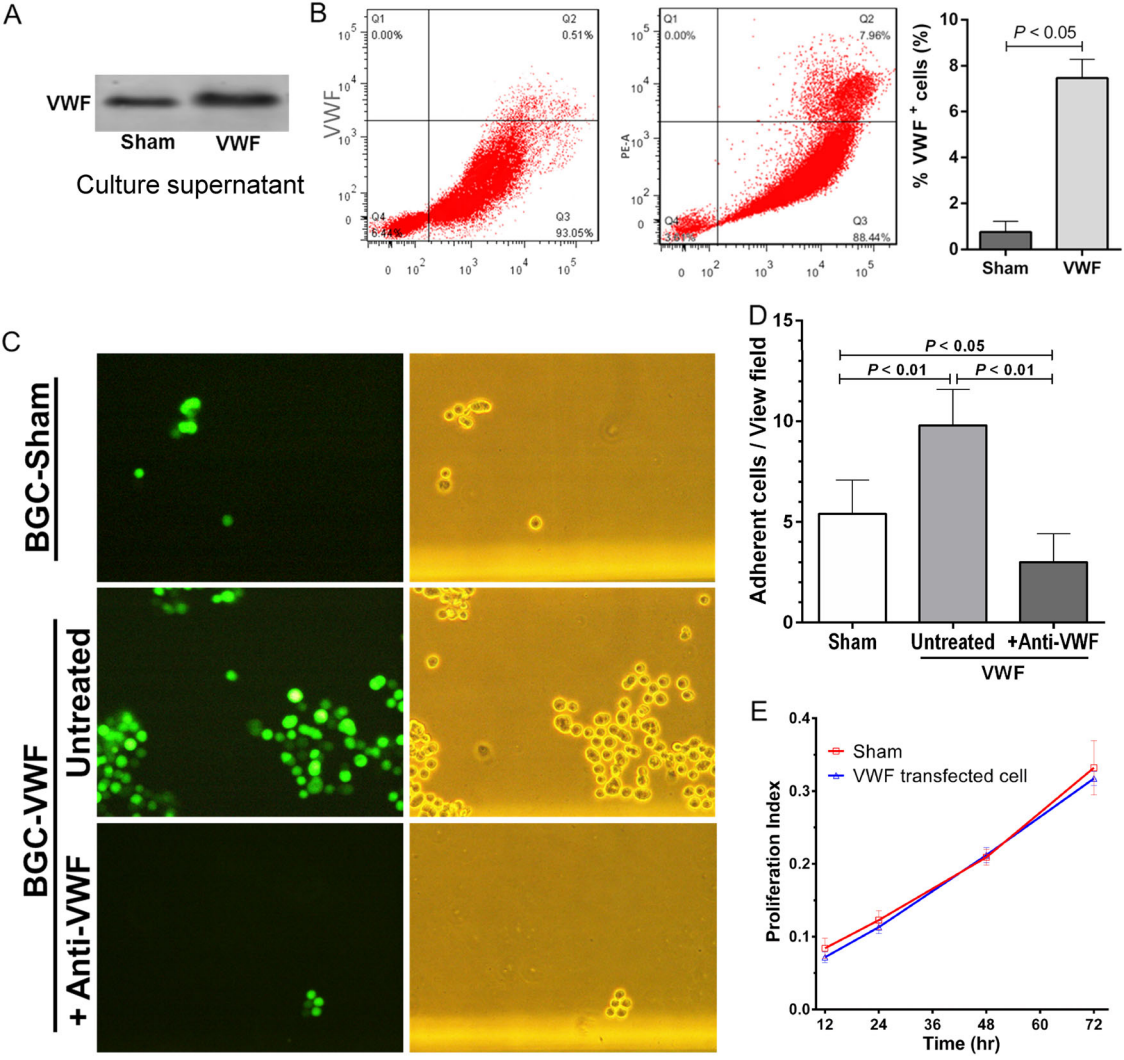
vWF overexpression enhances the adhesive activity of gastric cancer cells. **a** A representative vWF blot from the supernatants of vWF-transfected and sham-transfected cells from five separate experiments. **b** Flow cytometric detection of vWF on the surface of BGC823 cells transfected with human vWF cDNA or sham vector (*n* = 3, Student’s *t*-test). The values were after non-specific binding from an isotype control antibody was subtracted. **c** The representative images of sham-transfected and vWF-transfected BGC823 cells (that also expressed a GFP tracer) adherent to platelets under flow conditions in the presence and absence of an anti-vWF antibody (left panel is images of GFP^+^ cells and right panels is phase-contract images of the same cells). **d** A summary of data presented in C (*n* = 3, Student’s *t*-test). **e** BGC823 cells transfected with vWF-transfected and sham-transfected cells have similar rates of cell proliferation as measured by the MTT assay (*n* = 3, Student’s *t*-test)

We have shown that patients with poorly differentiated and more invasive gastric cancer had higher levels of plasma vWF (Figs. 1, 2). We have also shown that vWF secreted from cultured cancer cells was detected in solution and on cell surface. The surface-bound vWF could regulate the rate of metastasis by promoting cancer cells to self-aggregate and adhere to platelets and endothelial cells (Figs. 3, 4). To investigate this possibility, BGC823 cells were transfected with the human vWF cDNA to increase vWF expression (Supplemental Figure S1), because these clonal cells expressed less vWF than primary gastric cancer cells. The transfected cells secreted more vWF into the medium (Fig. 5a) and had more on their surfaces (Fig. 5b). vWF-overexpressing BGC823 cells increased their adhesion to platelets under venous blood flow (Fig. 5c) and the increased adhesion was blocked by an anti-vWF antibody (Fig. 5d). vWF-overexpression did not alter the rate of cell proliferation (Fig. 5e).

### vWF-mediated promotion of cancer metastasis in a mouse model

**Fig. 6:**
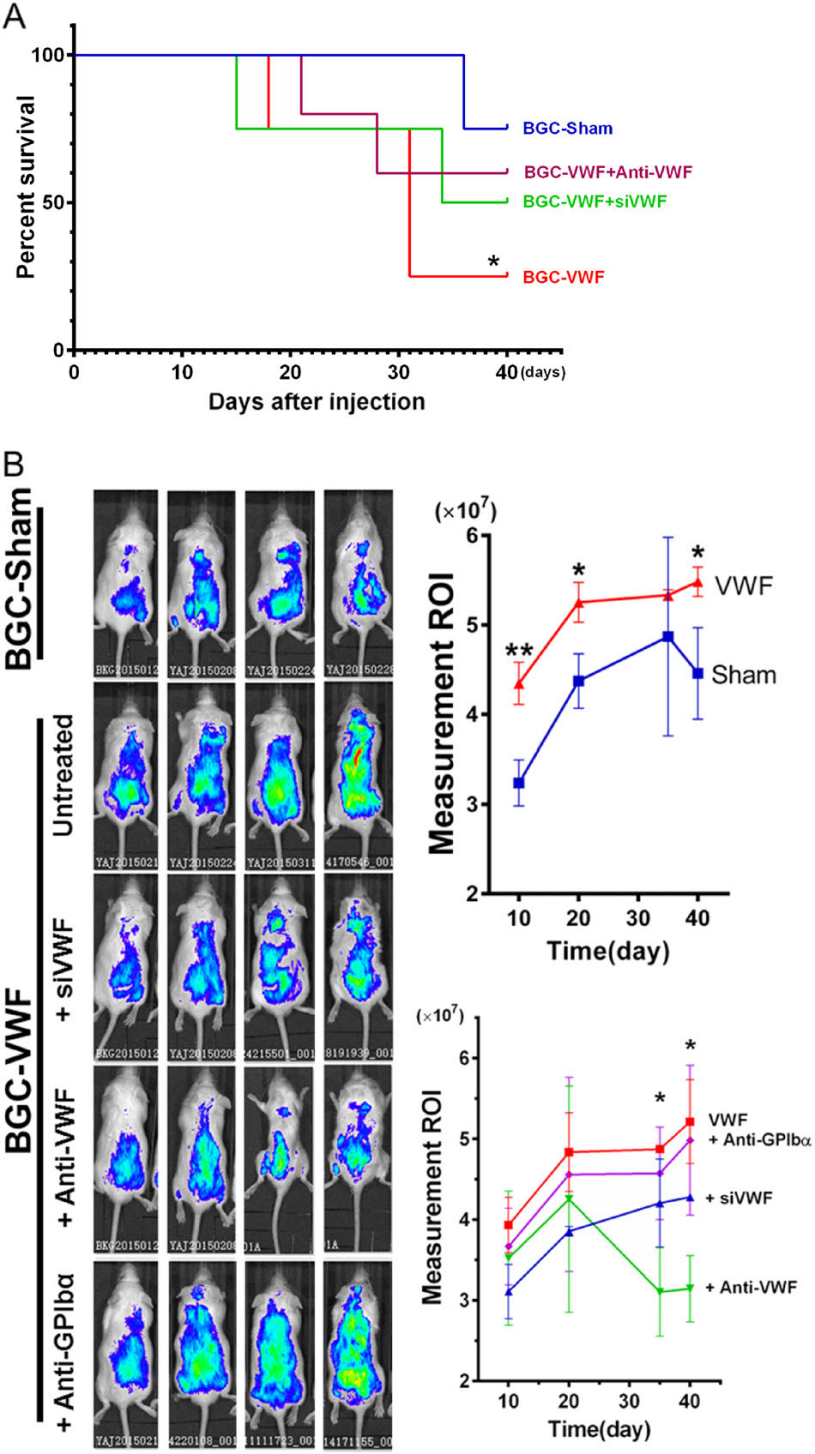
vWF promotes the cancer metastasis. **a** The Kaplan–Meier survival analysis of mice receiving vWF-transfected and sham-transfected BGC823 cells (*n* = 5, log-rank test). **b** The living image of mice 10, 20, 30, and 40 days after injection with vWF-overexpressing or sham-transfected BCG823 cells using the IVIS Lumina Imaging System (left panels). Mice also received vWF-overexpressing cells either pre-incubated with an anti-human vWF antibody or co-transfected with an inhibitory vWF siRNA. The right panels are summaries of the data from five independent experiments (*p < 0.05, **p < 0.01)

The results of in vitro experiments suggest a role of vWF in promoting BGC823 cells’ homotypic interaction among themselves and heterotypic interactions with platelets and endothelial cells (Figs. 4, 5). To test the hypothesis that these vWF-mediated interactions enhance cancer metastasis, we measured the pulmonary grafting of BGC823 cells that had either a basal or an enhanced expression of vWF. vWF-overexpressing and sham-transfected BGC823 cells were infused through the tail vein into NOD/SCID mice. We found that mice infused with vWF-overexpressing cells had a lower survival rate compared to parental BGC823 cells during a monitoring period of 40 days (Fig. 6a). The survival was significantly improved in mice infused with vWF-overexpressing cells that were either transfected with an inhibitory sivWF or pre-incubated with an anti-vWF antibody. Scans with an IVIS Lumina Imaging System showed that mice infused with vWF-overexpressing cells (that also expressed GFP as a tracer) had faster cancer grafts than those receiving sham-transfected cells (Fig. 6b). The enhanced grafts of BGC823 cells were blocked in mice receiving vWF-overexpressing cells that were transfected with the vWF siRNA (Fig. 6b). The graft enhancement was also blocked when the vWF-overexpressing cells were pre-incubated with either an antibody against human vWF, which targets only cancer cell-derived vWF or an antibody against both human and mouse CD42b, the platelet receptor for vWF (Fig. 6b, bottom right panel).

**Fig. 7:**
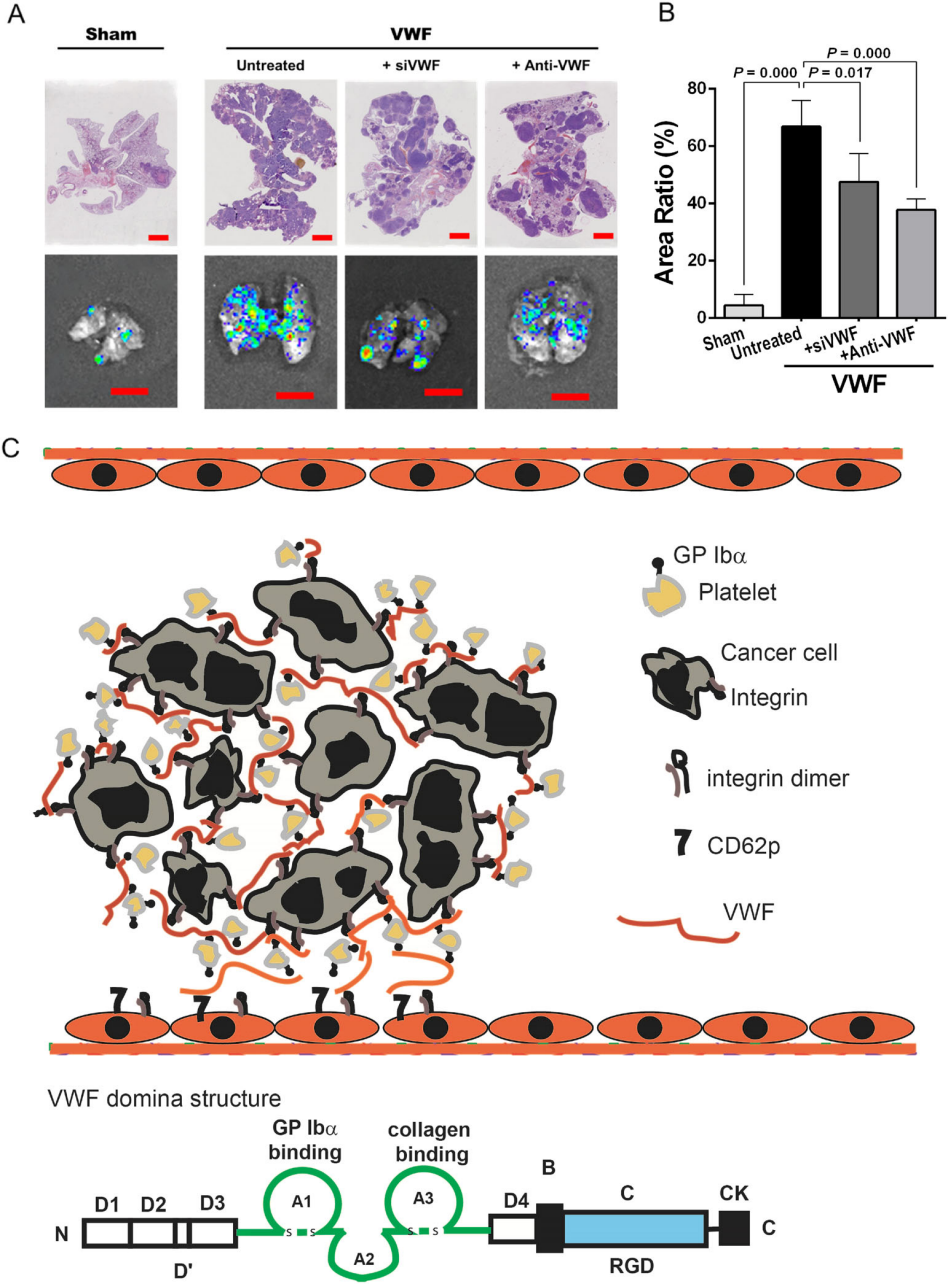
vWF-promoted cancer metastasis in mice. **a** The H&E stained histologic (top row) and fluorescent (bottom row) images of the lungs from mice receiving sham-transfected cells, vWF-overexpressing cells, and vWF-overexpressing cells that were either pretreated with a vWF antibody or co-transfected with a vWF-inhibitory siRNA (top panel, bar = 2000 μm, low panel, bar = 10 mm). **b** The summary of data from five independently tested mice (one-way ANOVA). **c** Schematic illustration of how cancer-cell-derived vWF could promote cancer metastasis through multiple pathways. First, large multimeric vWF can crosslink cancer cells to platelets through GP Ibα. Second, it increases plasma vWF antigen and reactivity to promote direct cancer cell interaction with vWF anchored to the endothelium. Third, cell-bound vWF also mediates cancer cell adhesion to resting and activated endothelial cells through integrins and CD62p, respectively

Consistent with the enhanced pulmonary grafts of BGC823 cells, the ratio of the tumor-bearing area to the total lung area was higher in mice receiving vWF-overexpressing cells than in those receiving sham-transfected cells (Fig. 7a). The vWF-inhibitory siRNA and the anti-vWF antibody also reversed the metastasis-promoting effect of vWF (Fig. 7b). Because there was no manipulation of the level of mouse plasma vWF, the data strongly implicated cancer cell-derived vWF in stimulating cancer metastasis. This experimental model was chosen specifically to investigate whether vWF promotes the extravagation of circulating cancer cells through endothelial cells of the vessel wall. It is not suitable for studying how cancer cells erode the subendothelial matrix to break into the circulation.

## Discussion

We have studied blood and tissue samples from gastric cancer patients and conducted experiments in vitro and in mouse models to investigate the role of vWF in regulating cancer metastasis. We made several novel observations that together define a critical role of vWF in promoting cancer metastasis.

First, vWF is expressed in primary gastric adenocarcinoma cells from patients (Fig. 1) and cells of the clonal gastric cancer BGC832 and MKN45 lines (Fig. 2), consistent with a recent report^29^. We further show that the clonal gastric cancer BGC823 cells secreted vWF without stimulation, but the secretion was enhanced by stimulating the cells with thrombin (Fig. 2). This pattern of basal and induced vWF secretions is similar to the constitutive and induced pathways of vWF secretion from endothelial cells. Consistent with the induced secretion of vWF, Weibel–Palade body-like structures similar to those found in endothelial cells were detected in BGC823 cells through electronic microscopy (Fig. 2f). Weibel–Palade bodies are endothelial cell granules, where ultra-large and more adhesive vWF multimers are stored and secreted through exocytosis induced by thrombin and inflammatory mediators^17,34^. The cancer cell-derived vWF provides a source of plasma vWF, in addition to endothelial cells that are the main source of vWF in the circulation^35,36^. More importantly, a substantial amount of vWF became cell-bound, being primarily mediated by β3 integrin (Fig. 4d), which has been demonstrated to express on the surface of cancer cells and to mediate cancer metastasis^37,38^. While the vWF receptor GP Ib-IX-V complex has been detected on human breast cancer cells^39^, the cancer-cell GP Ibα is unlikely to mediate the vWF binding to BGC823 cells because a β3 antibody completely removes cell-bound vWF (Fig. 4d). An interesting observation is that vWF immobilized onto BGC823 cells adheres to GP Ibα on platelets under a venous shear stress of 2 dynes/cm^2^ (Fig. 2g). We found that a CD42 antibody partially blocked the pulmonary metastasis of vWF-overexpressing BGC823 cells (Figs. 6b, 7a). The inhibitory effect of this antibody was less than that of anti-vWF antibody, probably because human vWF cross-reacts poorly to mouse CD42b. In this regard, one would expect platelets to play a greater role in promoting vWF-mediated cancer metastasis in patients with gastric cancer.

Second, the large multimeric structure of cell-bound vWF can mediate multiple cell–cell interactions simultaneously, as schematically illustrated in Fig. 7c. vWF can promote the homotypic aggregation of BGC823 cells by crosslinking β3 integrin (Fig. 3). Aggregated cancer cells have been shown to increase metastasis because they can evade attacks from the immune system^40,41^. Surface-bound vWF multimers can also link BGC823 cells to platelets through the RGD site in the C-domain and the GP Ibα binding site in the A1 domain (Figs. 4, 7c). This cancer cell-platelet complex can be captured to the endothelium through vWF interactions with CD62p^42–44^ and the integrin αvβ_3_^45^ on endothelial cells. vWF on cancer cells and on endothelial cells can also crosslink through lateral self-association^12,46^, which is highly resistant to mechanical forces exerted by the blood flow ^47^.

Third, the finding that vWF enhanced the homotypic interaction among cancer cells and heterotypic interactions with platelets and endothelial cells led to the hypothesis that vWF promotes blood-born cancer metastasis. This hypothesis was tested in a mouse model in which NOD/SCID mice were infused with vWF-overexpressing BGC823 cells and monitored for blood-borne cancer grafting. This model allowed us to specifically investigate whether cell-bound vWF promotes cancer cells to extravagate through the endothelium without the confounding influence of how cancer cells are released into the circulation. This model therefore has a limited value for studying how vWF can assist the release of cancer cells into the circulation by eroding the subendothelium and disrupting the endothelial cell barrier. Consistent with the in vitro observations, vWF-overexpressing BGC823 cells had a significantly greater ability to graft than sham-transfected cells, cells co-expressing a vWF-inhibitory RNA, or cells treated with either a vWF antibody or a CD42b antibody (Figs. 6 and 7). These data provide a mechanistic insight into why GP Ibα supports experimental lung metastasis of melanoma cells^48^ and highlight the importance of cancer-derived vWF. Our data further suggest that, although plasma vWF may have contributed to the formation of BCG823 cell aggregates, the pulmonary metastasis was primarily mediated by cancer-bound vWF. This is because BGC823 cells transfected with sivWF or treated with a vWF antibody were infused into mice after they were washed to remove free sivWF and antibody, and the rate of vWF synthesis and secretion from endothelial cells is expected to be identical in mice of all experimental groups.

In summary, we identified gastric cancer cells expressing vWF and demonstrated its critical role in promoting cancer metastasis through overlapping mechanisms (Fig. 7C). Our findings provide a new mechanistic insight into extensively documented clinical observations that associate an elevated level of plasma vWF with poor clinical outcomes for patients with cancer^16,22,25,28,49^. The findings also suggest that vWF is not only a biomarker^28,50,51^ but a mediator for cancer metastasis and a new therapeutic target for cancer.

## Materials and methods

### Patients

Patients diagnosed as having gastric cancer by histology were recruited to the study after they or their guardians provided written informed consent. Patients who received neoadjuvant chemotherapy or presented with confounding conditions known to increase plasma levels of vWF (diabetes, hypertension, heart failure, renal dysfunction, pregnancy, and hyperlipidemia) were excluded from the study. Healthy volunteers (*n* = 67, 19–71 years of age) were recruited as controls. Cancer tissues from endoscopic biopsy or surgery were collected and processed for vWF detection. Blood samples were also collected from the patients and controls (anticoagulant: 0.129 mol/L sodium citrate) and centrifuged at 700×*g* for 15 min at room temperature (RT) to collect plasma. This study was approved by the Lanzhou University Medical Ethics Committee on Conducting Human Research.

### Immunohistology, immunofluorescence, and flow cytometry

Paraffin-embedded tissues from biopsy or surgery were processed into 4-μm sections. After antigen retrieval in (Tris)-ethylenediaminetetra-acetic acid solution (pH 6.0, 95 °C for 40 min) and blocking of non-specific binding with non-immune serum, the sections were incubated with a rabbit anti-vWF antibody (Sigma-Aldrich, St. Louis, MO) overnight at 4 °C, then incubated with an HRP-conjugated goat anti-rabbit IgG (Sigma-Aldrich) for 2 h at RT. The peroxidase reaction was developed with 3,3′-Diaminobenzidine. For controls, the primary antibody was replaced by non-immune serum. All sections were evaluated by two independent pathologists with no prior knowledge of the patients.

For immunofluorescence, tissue sections or cultured cells from the gastric cancer lines BGC823 and MKN45, the osteosarcoma cell line (Saos2) and umbilical cord endothelial cells (HUVECs, China Center for Type Culture Collection [CCTC], Beijing) were fixed in 4% paraformaldehyde for 10 min at 4 °C and washed with phosphate-buffered saline (PBS). The fixed sections were blocked and permeabilized with 2% fetal calf serum, 2% bovine serum albumin (BSA), 0.1% Triton X-100, and 0.05% Tween-20 in PBS for 30 min at RT. They were then incubated with a mouse anti-human vWF antibody (Santa Cruz Biotechnology, Dallas, TX) overnight at 4 °C. After washing, the sections were incubated for 60 min with an FITC-conjugated anti-mouse IgG (Sigma-Aldrich) at RT and counter stained with DAPI (Thermo Fisher Scientifics, Waltham, MA) for 10 min at RT. Fluorescence images were captured using a Nikon Eclipse TE2000-U Inverted Microscope and analyzed with Image-Pro Plus 6.0 software.

For flow cytometry, BGC823 cells were detached with 1% trypsin, washed with PBS, and incubated with an anti-vWF antibody diluted in PBS containing 1% BSA for 40 min at RT (non-immune serum as control). The cells were washed with PBS and incubated with an Alexa Fluor 546-conjugated goat anti-mouse IgG for 30 min. The stained cells were analyzed on a FACSCalibur flow cytometer (Becton Dickinson, San Jose, CA). All values were presented after non-specific binding from non-immune serum had been subtracted.

### Immunoelectron microscopy

A pre-embedding immunogold staining method was used to detect intracellular vWF by electron microscopy^52^. Cells in suspension were fixed in a solution containing 2% paraformaldehyde, 2% glutaraldehyde, and 0.2% Triton X-100 for 5 h at 4 °C. They were then washed with PBS. After block non-specific binding with 1% BSA, the cells were incubated with a monoclonal anti-vWF antibody (Santa Cruz Biotechnology) at 4 °C overnight, and then washed with PBS and incubated for 2 h at RT with a goat anti-mouse IgG coupled to nanogold particles (Biosynthesis Biotechnology, Beijing). The cells were washed with PBS, sequentially fixed in 1% glutaraldehyde and 1% osmium tetroxide (30 min each), dehydrated in ethanol, and embedded in Epon 812 resin. Ultrathin sections (70–90 nm) were made, stained with 1% uranyl acetate, and observed under an electron microscope (JEM 1010, JEOL, Tokyo, Japan).

### DNA/siRNA transfection and real-time PCR

A full-length human vWF cDNA (Sangon Biotech Co. Ltd, Shanghai, China) was cloned into the mammalian expression vector pEGFP-N1 (Sangon Biotech), which also encodes a redshifted variant of green fluorescent protein (GFP). The DNA construct was transfected into the human gastric adenocarcinoma BGC823 cells (CCTCC) using Lipofectamine 2000 as the delivery reagent. Cells transfected with the pEGFP-N1 vector without the vWF cDNA and those without transfection served as controls. The transfected cells were cultured in a DMEM/F12 medium supplemented with 10% fetal bovine serum (GE Healthcare and Life Science, Logan, UT) and 500 μg/mL of the selection drug G418 at 37 °C for 14 days. Positive clones were isolated and cultured in the medium until confluence. The transfection was verified for GFP expression by flow cytometry (Beckman Coulter, Indianapolis, IN) and by immunoblots for vWF. For the inhibition experiments, vWF-overexpressing BGC823 cells were transiently transfected with an inhibitory vWF siRNA (Santa Cruz Biotechnology) according to the manufacturer’s instructions.

For real-time PCR, RNA was extracted from cells using TRIzol reagent (Thermo Fisher Scientific) and reversely transcribed into cDNA using Oligo(dT)15 primers. RT-PCR was performed using GoTaq Green Master Mix (Promega, Madison, WI). The cDNA template (2 μl) was added to 25 μl of a reaction buffer containing specific primers (Supplemental Table S1) and was amplified in the following steps: 95 °C for 2 min to denature DNA, 30 cycles of 94 °C for 30 s, 55 °C for 40 s, and 72 °C for 30 s to amplify DNA, and finally 72 °C for 5 min to anneal DNA.

### Immunoblots and ELISA

Cultured cells were detached (5 × 10^6^ cells/mL), washed with PBS, lysed in cold RIPA buffer for 5 min, sonicated for 30 s, and centrifuged at 14,000×*g* for 15 min to remove cell debris. The cell lysates standardized for the amount of protein by the Bradford method were separated through 8% polyacrylamide gel electrophoresis under reducing conditions and electro-transferred to a polyvinylidene difluoride membrane. The membrane was blocked with 5% skim milk in a Tris-HCl buffer (10 mM Tris-HCl, 150 mM NaCl, pH 7.6) containing 0.1% of Tween-20 at RT for 60 min, blotted with a vWF antibody overnight at 4 °C, and incubated with a second antibody coupled with an infrared dye for 60 min at RT. The antibody binding was detected using an Odyssey Infrared Imaging System (LI-COR Biosciences, Lincoln, NE). β-actin or glyceraldehyde 3-phosphate dehydrogenase (GAPDH) was used as a control for protein loading. A commercial ELISA kit was used to quantify vWF in blood samples from cancer patients and in culture media.

### Cell adhesion and aggregation assays

For cell adhesion assays, 96-well plates were seeded with washed human platelets for 2 h at 37 °C or with HUVECs that grew to confluence in a DMEM/F12 medium. BGC823 cells labeled with Hochest33342 (5 µg/mL, 10 min) were suspended in PBS and incubated with the following reagents: (1) none, (2) an anti-vWF antibody (1 μg/mL), (3) an anti-GP Ibα antibody (2 μg/mL), and (4) recombinant human vWF (0.5 μg/mL) for 30 min at 37 °C. The pretreated cells were added to platelet-coated or HUVEC-coated plates and incubated at 37 °C. After 20, 40, 60, 80, 100, and 120 min of incubation, the viability of cells adherent to platelets or HUVECs was evaluated with a commercial MTT colorimetric assay (Thermo Fisher Scientific). Cells adherent to HUVECs were also observed under the Nikon Eclipse TE2000-U Inverted Microscope for the Hochest33342 fluorescence. BGC823 cells that were transiently transfected with an inhibitory vWF siRNA were tested identically.

To measure cell adhesion to platelets under flowing conditions that mimicked blood flow, human platelet-rich plasma (PRP) infused into the Vena8 Fluoro + biochip microfluidic chambers (Cellix Ltd, Dublin, Ireland) was allowed to form a platelet matrix (30 min at RT). After being washed with PBS, BGC823 cells suspended in PBS were perfused through the chambers at 2 dynes/cm^2^ of shear stress for 30 min at RT. Cells adherent to the platelet matrix were recorded under Nikon Eclipse TE2000-U inverted fluorescence microscope.

For aggregation, cells (5 × 10^4^/mL) were suspended in DMEM/F12 medium containing 50% cell-free plasma (v/v), 50% platelet-rich plasma (PRP, v/v), 50% cell-free plasma with an anti-vWF antibody (10 μg/mL), or PRP with anti-vWF antibody (10 μg/mL). The cells were added to 6-well plates, gently rotated for 2 h at 37 °C, and then incubated for an additional 6 h without rotation at 37 °C. Cell aggregation was recorded under the Nikon Eclipse TE2000-U Microscope and analyzed using the Image-Pro Plus 6.0 software.

### MTT cell proliferation assay

Cells were seeded at 5 × 10^2^ cells/well in 96-well plates and cultured in DMEM/F12 medium. After 12, 24, 48, and 72 h in culture, they were incubated with MTT (5 mg/mL) for 4 h at 37 °C and shocked with 150 µL DMSO for 10 min at RT. The optical density (OD) of the cells was immediately measured using a Bio-Rad 680 microplate reader (Bio-Rad Laboratories, Hercules, CA) at OD490 nm.

### Mouse models of blood-born cancer metastasis

All procedures were performed according to the Guidelines for Animal Care and the Use of Laboratory Animals of Lanzhou University. Six-week-old male nonobese diabetic and severe combined immune deficient mice (NOD/SCID, HFK Bioscience, Beijing) were randomly divided into five groups, anesthetized with isoflurane, and infused through tail veins with 4 × 10^6^/300 μL of the following cells: (1) parental BGC823 cells, (2) sham-transfected BGC823 cells, (3) BGC823 cells transfected with human vWF, (4) vWF-transfected BGC823 cells that were subsequently transfected with an inhibitory vWF siRNA, (5) vWF-transfected BGC823 cells treated with an anti-vWF antibody (Sigma-Aldrich), and (6) vWF-transfected cells treated with a polyclonal anti-CD42b antibody (Santa Cruz Biotechnologies). The cancer metastasis was monitored with the IVIS Lumina Imaging System every 10 days after the mice were anesthetized with isoflurane. The photon data were analyzed using Living Image software. At the end of the monitoring period, the mice were killed and their lungs collected for pulmonary cancer grafts using the IVIS System. After scanning, the lungs were fixed with 4% paraformaldehyde, embedded in paraffin, sectioned, and stained with hematoxylin and eosin (H&E). Serial H&E sections were scanned with a Leica SCN400 digital slide scanner to digitize tissue images, which were used to calculate the ratio of cancer area to the total area of the lungs, as a measure of pulmonary cancer metastasis.

### Statistics

The data are presented as means ± standard deviation (SD). Statistical analyses between two samples and among multiple samples were performed using Student’s *t*-test and one-way analysis of variance (ANOVA) with Tukey test, respectively using the SPSS Statistics 21 program (IBM, NY). Pearson’s correlation coefficient was used to measure the relationships among the variables. Kaplan–Meier log-rank survival analysis was performed to evaluate mouse survival after cancer grafts. A *p*-value of <0.05 was considered to be statistically significant.

## Electronic supplementary material

Supplemental Material
