# Supplementary material for: Cancer cell-derived von Willebrand factor enhanced metastasis of gastric adenocarcinoma
Source: Oncogenesis. 2018 Jan 24;7(1):12. doi: 10.1038/s41389-017-0023-5 (PMC5833464; doi:10.1038/s41389-017-0023-5)
Supplement: Supplementary file 1 — Supplemental Material [file 41389_2017_23_MOESM1_ESM.docx]

**Supplemental Table S1: The sequence-specific primers designed for RT-PCR***

| **Primers** | **Forward** | **Reverse** |
| --- | --- | --- |
| VWF | 5'-TCGGACCCTTATGACTTTGC-3' | 5'-TACAGCACCATTCCCTCCTG-3' |
| β-actin | 5'-TGACGTGGACATCCGCAAAG-3' | 5'-CTGGAAGGTGGACAGCGAGG-3' |

*The primers were synthesized by Sangon Biotech, Co. Ltd. Shanghai, China)

**Supplemental Figure S1**


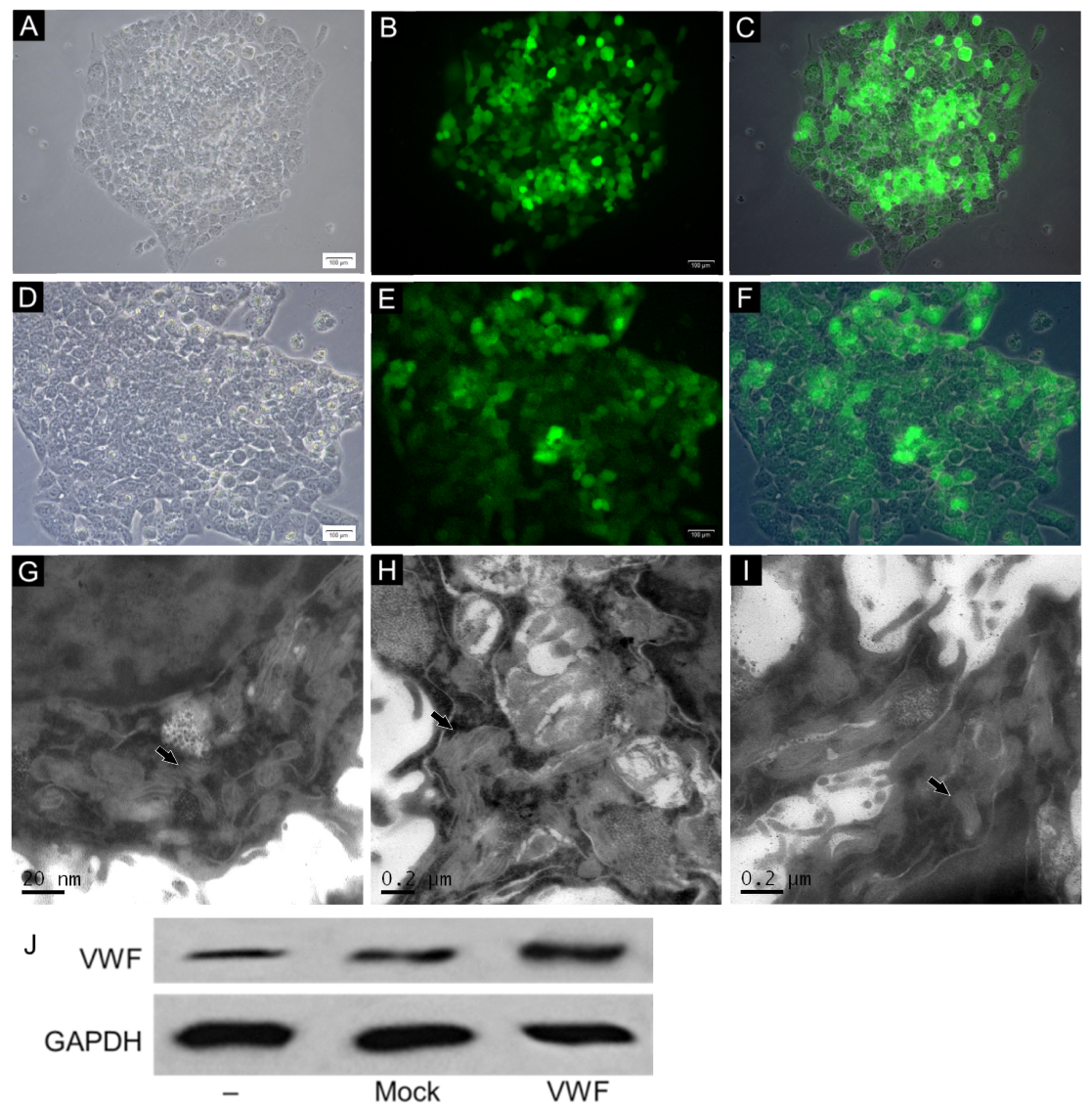


**Supplemental Figure S1: Structured VWF-overexpressing cells.** BGC823 cells stably transfected with human VWF cDNA (A-C) or vector along (D-F), positive colon was screen out. Western blotting detection showed the VWF expression enhanced in BGC823 cells transfected VWF gene (J). The transmission electron microscope(TEM) observation showed WPBs-like structure in BGC823 cells transfected VWF gene which were trypsinized in single cell (G), or in sheets cells (H) and scraped cells (I).( bar = 100μm)
